# Challenges of Newborn Hearing Screening Programs in Saudi Arabia: A Systematic Review

**DOI:** 10.3390/audiolres15020034

**Published:** 2025-03-25

**Authors:** Ahmad A. Alanazi, Nannette Nicholson

**Affiliations:** 1 Department of Audiology and Speech Pathology, College of Applied Medical Sciences, King Saud Bin Abdulaziz University for Health Sciences, P.O. Box 3660, Riyadh 11481, Saudi Arabia; 2 King Abdullah International Medical Research Center, Riyadh 11481, Saudi Arabia; 3 NorthStar Audiology, Colorado Springs, CO 80939, USA

**Keywords:** audiology, challenges, hearing, newborns, neonates, Saudi Arabia, review, screening

## Abstract

Background/Objectives: Although newborn hearing screening (NHS) programs have been successfully implemented in many countries worldwide, challenges to these programs have been reported in the literature, such as a lack of awareness among families and healthcare professionals and limited funding. Challenges to the NHS programs in Saudi Arabia have not been systematically reported. This study aimed to assess the level and quality of evidence supporting the existing challenges of the NHS programs in Saudi Arabia. Methods: A systematic review of all peer-reviewed literature on Saudi NHS programs published between 2016 and 2024 was conducted according to the PRISMA guidelines. Search strategies were executed in seven databases. Data were collected from studies that met the inclusion criteria. Results: A total of 37 records were reviewed, and 13 peer-reviewed publications met the inclusion criteria. Characteristics of included studies were classified by study language, study sample, sample size, location of the study sample, study purpose, and study method via evidence tables. Each study was critically appraised according to the quality. Results revealed that cross-sectional prospective description was the main research design with low strength of evidence. Six main challenges were identified and described: lack of awareness and gaps in knowledge, lost to follow-up, inadequate data entry, management, and tracking system, limited or absence of services in some residential areas, insufficient training and frequent changes of NHS personnel, and absence of a standardized NHS protocol. Conclusions: This review may assist in overcoming these challenges and improving the NHS programs in Saudi Arabia. There is a need to establish national education campaigns about the NHS programs, improve documentation by using database management and tracking systems, and expand the early hearing detection and intervention (EHDI) services for children in all Saudi regions.

## 1. Introduction

Hearing plays an important role in learning spoken language. The development of language is strongly related to cognitive, social, and emotional development [1,2]. Hearing loss can have significant negative effects on language development, speech, and cognition. These effects can then have an impact on future academic and career opportunities, as well as mental health and social interactions [3,4,5]. According to the World Health Organization (WHO), approximately 430 million people worldwide, including 34 million children, have disabling hearing loss and require rehabilitation [6]. Congenital hearing loss is one of the most chronic conditions in children that affects 2–3 in every 1000 births [7]. Varying prevalences of both permanent and fluctuating hearing loss among Saudi children were reported in the literature ranging from 1.75% to 13% [8,9,10]. Saudi children commonly suffer from both conductive hearing loss and sensorineural hearing loss (SNHL) [11,12]. Without appropriate intervention, the negative impact of hearing loss in children continues into later life [13].

Early hearing detection and intervention (EHDI) programs have been established to ensure adequate access to linguistic stimulation and intervention services as quickly as possible by screening newborns before one month of age, completing audiologic diagnosis before three months of age, and providing early intervention before six months of age (i.e., the 1–3–6 EHDI timeline) [14]. The Joint Committee on Infants Hearing (JCIH) recommends the EHDI programs that have met this timeline set a new target of 1–2–3 months (screen hearing of newborns before one month of age, diagnose before two months of age, and provide early intervention before three months of age) [14]. The positive outcomes of the EHDI program have been well documented in the literature [3,15]. The first critical step of the EHDI program is newborn or neonatal hearing screening (NHS).

The process of NHS involves simple and non-invasive tests to assess the auditory system of newborns. By detecting hearing loss early, audiologists and other healthcare professionals can initiate timely interventions and provide appropriate support to maximize a child’s communication and language development. There has been a growing recognition of the importance of NHS [16]. The WHO recommends all countries adopt NHS protocols and establish appropriate rehabilitation services [17], so several countries have taken significant steps to implement universal NHS programs [16].

In Saudi Arabia, the collaboration between the Ministry of Health (MOH) and the Saudi Association for Hearing Impairment led to the launching of the first NHS program in two hospitals located in Riyadh in 2007 [18]. Before that, hospitals that were not under the MOH administration, such as military hospitals and private hospitals, had implemented their own NHS programs. In 2014, the government legislated hearing screenings for all newborns in Saudi Arabia that are covered either by the government or insurance companies [18,19]. In 2016, the MOH started the first phase of the NHS program, covering more than 60% of newborns in 30 referral hospitals to screen every newborn within 72 h of birth [20]. The current coverage rate of NHS is more than 96% with a refer rate of 0.7% in the MOH hospitals across Saudi Arabia [19]. Despite these efforts, challenges, and barriers, such as lack of awareness among parents and healthcare professionals, lack of infrastructure, inadequate funding, and scarcity of qualified professionals may hinder the effective implementation of NHS programs [21]. Unfortunately, there has been no research specifically designed to explore the up-to-date challenges to the NHS programs in Saudi Arabia. Therefore, this systematic review asked the following question: what are the challenges faced by the NHS programs in Saudi Arabia? Our study aimed to explore these challenges and provide recommendations for improvement.

## 2. Materials and Methods

This systematic review was approved by the Institutional Review Board (IRB) of King Saud bin Abdulaziz University for Health Sciences protocol number (IRB/NRR24/017/4).

### 2.1. Search Strategy

The procedure for Preferred Reporting Items for Systematic Reviews and Meta-analyses (PRISMA) is recommended to report the methods and findings of systematic reviews [22]. Our study adheres to the PRISMA guidelines to maintain transparency, comprehensiveness, and methodological rigor in reporting our systematic review. We have utilized the PRISMA checklist and flow diagram to outline the processes of study identification, screening, eligibility assessment, and inclusion, thereby strengthening the reproducibility and reliability of our findings. The following electronic databases, PubMed, ScienceDirect, CINAHL via EBSCOhost, Cochrane Central, Embase, and Arab World Research Source, were searched in November 2024. The bibliographies of recent literature reviews and currently reviewed articles, as well as Google Scholar, were used to find relevant publications. The search results include all relevant citations that were found. Keywords, syntax terms, and Boolean phrases, such as (newborn hearing screening OR neonatal hearing screening OR infant hearing screening) AND (Saudi Arabia) AND (challenges OR difficulties OR threats) were used to retrieve available articles from these databases. Appendix A shows an example of PubMed search terms and strings.

### 2.2. Inclusion and Exclusion Criteria

Research studies conducted to investigate NHS and its related aspects including challenges in Saudi Arabia were included. The inclusion criteria contained all study designs. Studies that were available electronically in peer-reviewed journals and published in both Arabic and English were included. Studies that were published before 2016 were excluded because the MOH, which oversees the NHS programs in the MOH birth hospitals in Saudi Arabia, started the first phase of the NHS program in 2016. Therefore, this systematic review included studies published between 2016 and 2024, ensuring a comprehensive analysis of recent research within this timeframe.

### 2.3. Eligibility and Selection

**Figure 1:**
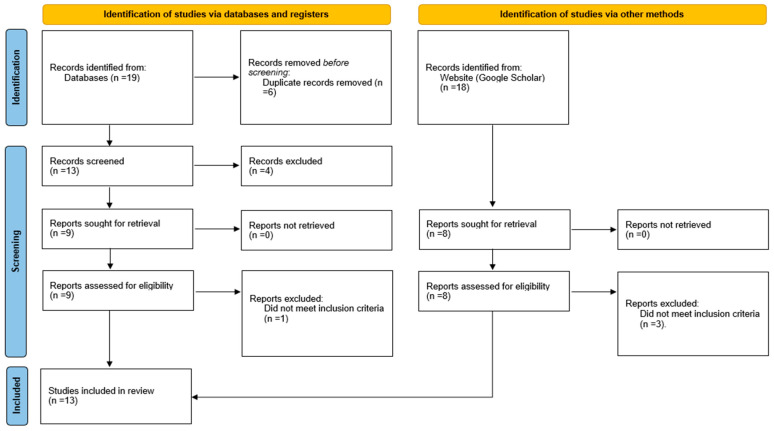
PRISMA flow diagram demonstrates the search process. Adapted from Page et al. [22].

A detailed description of the systematic review’s search plan and selection criteria was developed. Initially, the search strategy was implemented and completed. Second, the papers’ titles were checked for relevancy; any research judged irrelevant did not receive further consideration. Third, the abstracts of the remaining papers were retrieved electronically, and their applicability was evaluated. The studies that met the requirements for inclusion were subsequently downloaded and printed out for careful examination. The authors assessed each study’s eligibility and methodological quality twice to ensure no bias in the publications they chose to include or reject. The PRISMA flow diagram was used to guide this procedure (Figure 1).

### 2.4. Critical Appraisal

Two authors evaluated the studies that were chosen for retrieval independently. Every author scored every study on their own. Critical assessment ratings were compared, and disagreements were discussed and resolved. Following the establishment of consensus, the strength of evidence of the included studies was evaluated.

### 2.5. Strength of Evidence

Each of the studies meeting the inclusion criteria was also rated for quality (strength) of evidence and included three categories (High, Moderate, Low). The strength of evidence classification does not mean that studies rated as having moderate or low strength of evidence are not useful, but it is essential for guiding decisions in evidence-based practices.

1. High: Random assignment studies with low attrition of sample members and no reassignment of sample members after the original random assignments.
2. Moderate: Random assignment studies that, because of flaws in the study design, execution, or analysis, do not meet all the criteria for the high rating; matched comparison group designs that establish baseline equivalence on selected measures; and single case and regression discontinuity designs.
3. Low: Impact studies that do not meet the criteria for high or moderate.

### 2.6. Data Extraction and Synthesis

Data extraction and synthesis were performed on all studies, regardless of the quality of their methodology. An Excel spreadsheet was developed by the authors to extract data. The data extraction contained details on the included studies and their findings that were relevant to this review. The data were organized into evidence summary tables, and a narrative explanation of the outcomes was provided.

## 3. Results

The identification and selection of the studies included in this systematic review were represented by the PRISMA flow diagram (Figure 1). The initial search strategy identified 37 publications from databases and other sources. Ten duplicates were removed, leaving 27 publications for title and abstract review. The number of publications for each source after duplicate removal is shown in Table 1. Ten publications were excluded due to irrelevance based on the title and abstract review. A full study review was undertaken on the remaining 17 publications to determine eligibility based on inclusion and exclusion criteria. This process identified 13 studies meeting the study criteria. These publications were subjected to data extraction and analysis of results.

### 3.1. Study Characteristics

The study language, study sample, sample size, location of study sample, study purpose, and study method varied among the included studies (Table 2). In this systematic review, all the studies (N = 13) were published in the English language [18,19,23,24,25,26,27,28,29,30,31,32,33] The study sample involved newborns (n = 5) [18,19,23,25,31], parents/caregivers (n = 7) [24,25,26,29,30,31,33], pediatricians (n = 2) [27,32], and family physicians (n = 1) [28]. The range of sample size in the current systematic review was 1166 to more than one million for newborns, 60 to 1533 for parents, and 67 to 216 for healthcare professionals. The variation in ranges depended on the study method and what data registry was searched. Most studies included samples from all Saudi regions (6 of 13 studies) [19,23,24,26,28,32]. The remaining studies were conducted in Riyadh (n = 3) [18,25,33], Jeddah (n = 1) [27], Riyadh and Dammam (n = 1) [31], Qassim (n = 1) [29], and AlAhsa (n = 1) [30]. Based on the type of sample, most of the studies reported the sample characteristics, such as gender, age, educational level of parents, number of children, monthly income, and years of experience for healthcare professionals. The purpose of conducting the included studies was to examine knowledge, attitudes, and perceptions regarding NHS programs, EHDI services, and hearing loss risk factors (n = 7) [24,26,27,28,29,30,32], investigate rates, such as coverage rate, lost to follow-up (i.e., it indicates that the infant did not receive the recommended diagnosis or treatment) rate, and age of hearing loss identification rate (n = 4) [18,23,25,31], and generally evaluate the status of NHS and early intervention services (n = 2) [19,23]. Data were collected by searching the registries (n = 4) [18,23,25,31], distributing validated questionnaires (n = 8) [24,26,27,28,29,30,32,33], interviewing the target population (n = 2) [25,31], and reviewing the scientific evidence (n = 1) [19].

### 3.2. Strength of Evidence

The research design and the strength of evidence are shown in Table 3. The common study designs used for achieving the aims of the included studies were cross-sectional descriptive study designs that were either prospective (n = 8), retrospective (n = 2), or combining both (n = 2). The strength of evidence for all the included studies was classified as low.

### 3.3. Challenges of the NHS Programs

Table 4 summarizes several challenges reported by the included studies that may negatively affect the effectiveness of the NHS programs in Saudi Arabia. It is worth mentioning that not all the included studies directly examined the challenges of the NHS programs; however, some of these challenges were reported as recommendations for improving the NHS programs. The challenges of the NHS programs were as follows: (a) lack of awareness and gaps in knowledge, (b) lost to follow-up, (c) inadequate data entry, management, and tracking system, (d) limited or absence of services in some residential areas, (e) insufficient training and frequent changes of NHS personnel, and (f) absence of a standardized NHS protocol.

Lack of awareness and gaps in knowledge among parents (n = 10) and healthcare professionals (n = 3) were the main reported challenges [18,19,23,24,25,26,27,28,29,30,31,32,33]. Lost to follow-up was also reported as a major challenge (n = 4) because of several parental and logistic reasons (e.g., failure to remember and unavailable transportation) [18,19,23,25]. Inadequate data entry, management, and tracking system (n = 4), such as not including the diagnostic stage and NHS data of non-MOH and private hospitals in the NHS national registry and the absence of automated data entry, is another challenge [18,19,23,25]. Lack of services in some residential areas that may delay meeting the recommended EHDI timeline (n = 4) was stated as a challenge in the included studies [18,19,31,33]. The other challenge is insufficient training for NHS personnel (n = 3), particularly for effective counseling [18,19,33], and frequent changes in the NHS team, especially trained nurses [19]. Another included study showed that the absence of a standardized NHS protocol among all governmental and non-governmental hospitals was a challenge [18].

## 4. Discussion

The NHS programs were successfully implemented in Saudi Arabia because of careful planning before the program began and the use of advanced technologies [19]. However, this systematic review showed some challenges of the NHS programs in Saudi Arabia.

### 4.1. Challenges

#### 4.1.1. Lack of Awareness and Gaps in Knowledge

Ten studies in the current systematic review showed that the lack of parental awareness of NHS and EHDI is a challenge to the NHS programs [18,19,23,24,25,26,29,30,31,33], while three studies revealed that the existing gap in knowledge among healthcare providers (pediatricians and family physicians) is another challenge to the NHS programs [27,28,32]. Scheepers et al. reported that the most common causes for refusing hearing screening are the caregiver’s knowledge of the screening, healthcare professionals’ knowledge and team collaboration, and costs [34]. Pynnonen et al. identified the absence of parental awareness of NHS across the United States [35]. Deficiencies in knowledge about NHS and hearing loss among physicians were also reported in the literature [36,37].

Parental awareness is a major factor in determining the NHS performance and has a direct association with the lost to follow-up rate [18]. Collaborative relationships between parents and healthcare professionals are essential. Parents and families are important team members. The role of parents in the 1–3–6 EHDI timeline is considered a critical component in the family-centered care approach, in which healthcare professionals address the needs of both the patient and his or her family members [38]. Since the patient is an infant, the parents are the family members who are involved in all aspects of clinical care. Hanft et al. reported that this care model requires professionals to inform and support families to make adequate decisions for their children [38]. The family-centered care philosophy and practice improves intervention services, patient’s health condition, patient satisfaction, and family behavior [39,40].

In the United States, the majority of children who are deaf (90–95%) are born to hearing parents [41]. In Saudi Arabia, consanguineous marriage causes a variety of hereditary progressive cochleovestibular malformations, which are linked to both syndromic and non-syndromic hereditary hearing loss [18]. Hearing parents may have different negative emotions (e.g., guilt, anger, confusion, disappointment, and stress) once their child is identified with hearing loss [42,43]. They may also know little or nothing about hearing loss and its consequences and have little to no experience making decisions regarding the choice of communication methods (e.g., spoken language versus signed language), amplification devices, and educational environments [41,43].

The success of children with hearing loss is affected by parents’ education, attitudes (e.g., reactions and acceptance), and encouragement of their child [44]. Parents should be well informed and included in this collaborative work to help them make knowledgeable decisions about their child’s hearing loss. Furthermore, the education about NHS should be extended to include healthcare professionals who have much misinformation regarding the ability to test infant hearing loss and the importance of NHS and follow-up appointments [45]. This misunderstanding could be more pronounced or apparent when there is insufficient equipment, weak training, and a scarcity of qualified personnel [46]. Healthcare professionals should be aware of the NHS program, knowledgeable about the technology used to screen infant hearing, and mindful of the 1–3–6 EHDI timeline.

#### 4.1.2. Lost to Follow-Up

A total of 4 out of 13 included studies reported that lost to follow-up is a challenge to the NHS [18,19,23,25]. Poor follow-up return rate was reported as a challenge to the NHS programs in both developed and less developed countries [34,47,48]. In Saudi Arabia, the lost to follow-up rate is varied. One study estimated the lost to the system (i.e., the combination of lost to follow-up and lost to documentation) at 34.9% [18], while another study estimated the lost to follow-up rate at 18% [25]. As reported earlier, lost to follow-up indicates that the infant did not receive or complete the recommended diagnostic or intervention process [18], while lost to documentation means “infants who did not pass their hearing screening and whose diagnostic or intervention status has not been reported to the EHDI program; thus, their status remains unknown by the EHDI program despite the fact that they may have received services” [49].

According to the Centers for Disease Control and Prevention (CDC), approximately 27.5% of newborns referred for diagnosis following delivery of hearing screen results were lost to follow-up [47]. Parental awareness of the importance of NHS-subsequent follow-up appointments is critical. The primary cause of missing follow-up appointments in Saudi Arabia was stated to be the parental lack of awareness of NHS and follow-up appointments [18,25]. This necessitates rigorous monitoring and follow-up procedures after the first stage of NHS [14]. Increased costs and lack of parental knowledge of the next step following NHS were reported to be the reasons for NHS refusal in South Africa [34]. Parents are not required to pay for NHS because it is covered by the government or medical insurance in Saudi Arabia [19]. Other parental reasons may increase the lost to follow-up rate in Saudi Arabia, such as the lack of awareness, failure to remember, parental health conditions, absence of transportation, and work commitments [25]. Lack of adequate tracking technology may make it difficult to monitor all newborns’ hearing screening data and follow those who did not pass the initial NHS or who have risk factors [50].

#### 4.1.3. Inadequate Data Entry, Management, and Tracking System

Four included studies reported the existence of inadequate data entry, management, and tracking systems as challenges to the NHS programs [18,19,23,25]. The quality of many NHS programs is compromised by the absence of structured and organized databases and data gathering. According to the survey data of individuals involved with the NHS programs in 196 countries, there were no tracking procedures in place for babies who were not screened or for those who did not pass the screening and would need to be sent for audiological diagnosis and treatment services [51]. The lost to follow-up rate is typically high or unknowable in the absence of tracking [51]. The absence of tracking and data collection methods is one factor contributing to the ineffectiveness of follow-up procedures that can be avoided by using appropriate data management systems [52]. Furthermore, frequent data analysis on screening coverage and failure rates should be used as process indicators for the NHS program quality control [53].

In the United States, the EHDI Information System (EHDI-IS) is a tool available in every state and territory used to (a) assist programs in ensuring that all infants receive follow-up services in compliance with the 1–3–6 EHDI timeline, (b) gather data on the prevalence of infants with hearing loss, and (c) provide a variety of relevant data analysis that assists in tracking, surveillance, and assessment of program performance [54]. Alanazi recommended establishing a follow-up center and computer tracking system in Saudi Arabia to reach and notify parents a few days before the recommended visit and use automatic transfer of data from the screeners to the database without being manually inputted [18]. The NHS programs are also recommended to include all data of NHS that is conducted in all hospitals, governmental (whether MOH or non-MOH hospitals) and private, under one national registry [23]. Also, this registry is advised to include the results of the diagnostic stage. The integration of telemedicine components and bidirectional data flow between NHS centers and decentralized screening devices should be considered [51].

#### 4.1.4. Lack of Services in Some Residential Areas

A total of 4 out of 13 included studies reported the absence of services in some Saudi residential areas which may delay meeting the recommended EHDI timeline [18,19,31,33]. The extent to which the EHDI programs are implemented and covered varies widely between countries and even within the regions of the same country [55]. The NHS program has not been implemented for all newborns in the world, particularly in rural areas of poor nations where the required equipment is lacking [56]. For example, half of the European countries have implemented the EHDI programs nationwide, whereas almost all the countries in the Southeast Asia region have not established any NHS programs [55].

The national NHS program has been legislated by the government for all newborns in Saudi Arabia, and the coverage rate of NHS has increased since 2014 [19]. Alkahtani et al. stated no nationwide covering of the NHS program in Saudi Arabia [31]. Alaql reported that the coverage rate of NHS is more than 96% in the MOH hospitals across Saudi Arabia [19]. Alothman et al. estimated the coverage rate of NHS at 92.6% in Saudi Arabia [23]. According to the CDC, the coverage rate of NHS was reported to be 98.4% in the United States [47]. Alyami et al. attributed the delay of EHDI services to the distribution of these services mainly in metropolitan areas, such as Riyadh [33]. The average age of cochlear implantation is 45.7 months in Saudi Arabia, while it is 21.5 months in the United States [57]. Traveling long distances to hospitals where the EHDI services exist is costly for some families, who may need financial assistance [35]. Such challenges were reported in the literature where families face transportation and financial difficulties to reach the EHDI services that existed in only some hospitals [58,59]. Alanazi recommended the expansion of the EHDI services, not only NHS, in all Saudi cities [18].

#### 4.1.5. Insufficient Training and Frequent Changes in the NHS Personnel

Three studies identified the existing insufficient training, particularly effective counseling, for NHS personnel and the frequent change in NHS staff [18,19,33]. The success of the NHS program and the EHDI services generally depends on training healthcare professionals on both technical and communication skills. Healthcare professionals are responsible for guiding parents regarding the outcomes and the following steps in the process. There are a limited number of professionals with knowledge and experience in this field [48]. One factor that affects the standard of patient care in audiology is informational counseling for parents and/or patients [60]. Revealing to parents that their child has hearing loss is a part of the counseling process that depends on breaking bad news. The clinicians must be able to handle this type of situation and provide parents with emotional support.

NHS is mainly performed by nurses who work in well-baby nurseries and neonatal intensive care units; therefore, they play a crucial role as the point of contact for promoting hearing screening, providing parental counseling, and ensuring follow-up care [61]. However, several studies evaluating nurses’ attitudes, knowledge, and behaviors on NHS and early intervention found that nurses often did not know about the various screening protocols, phases of referral, risk factors for hearing loss, or the availability of resources [62,63,64]. This systematic review revealed the existing gaps in knowledge about the NHS programs and the EHDI services among pediatricians and family physicians [27,28,32]. Several studies showed a low level of knowledge about NHS among physicians [65,66]. This gap in knowledge among healthcare professionals could be a result of the lack of appropriate training. Besides audiologists, family physicians, pediatricians, and nurses are on the frontline in dealing with hearing-impaired children and their families. They are responsible for helping parents when breaking bad news if the child did not pass NHS, informing them about the diagnosis and next-step recommendations, and providing take-home information. Through the use of simulation, Alanazi et al. created a curriculum for training on technical and counseling skills related to NHS [67]. Training healthcare professionals is crucial in the process of successfully employing and implementing the NHS program and the 1–3–6 EHDI timeline.

One of the included studies reported that frequent changes in the NHS team, especially trained nurses, may affect the success of the NHS program [19]. Two main barriers to maintaining effective teamwork are the instability of teams and changing roles [68]. The NHS team must be stable because it is not like other healthcare teams, such as trauma teams that are formed for a temporary task. If a new team member joins the NHS program, proper training should be provided. Furthermore, the roles and responsibilities of the NHS team should be clear without any overlap to achieve the effectiveness of the NHS program.

#### 4.1.6. Absence of a Standardized NHS Protocol

One of the included studies reported no unification of the test protocol between governmental and non-governmental hospitals [18]. Typically, the NHS program is a two-phase approach. Before being discharged from the hospital, a newborn is screened in the first few days of life utilizing automated auditory brainstem response (AABR) and/or otoacoustic emissions (OAEs). Both AABR and OAE technologies were recognized as optimal tools for NHS and hearing loss diagnosis. AABR and OAE screeners do not require behavioral responses from the infant or demand interpretation by the hearing screening personnel. This means both hearing screening technologies show either pass or fail/refer, which is ideal for non-audiologist personnel, such as nurses. The newborns will be referred for rescreening (second stage) if they do not pass the initial round within a few days or weeks [18]. This screening includes either one or two phases of OAEs testing, or both OAEs and AABR testing in high-risk cases [69]. The NHS program may follow a three-stage screening strategy, which was suggested to reduce the referral rate [19,25]. The NHS programs at the MOH adopted three AABR screenings for all newborns in Saudi Arabia [18]. While the NHS programs funded by the MOH implements the AABR screening [19], some private hospitals still utilize OAEs. It is recommended to use tympanometry, OAEs, and frequency-specific ABR testing to evaluate the infant’s hearing [18,49].

### 4.2. Alignment with Global NHS Challenges and Future Research Directions

The NHS programs in Saudi Arabia encounter some challenges that are consistent with global experiences. A significant barrier to the success of NHS programs is the lack of awareness and knowledge among parents. In Saudi Arabia, the included studies in the current systematic review have shown that there are still gaps in knowledge that require further investigation and intervention [18,19,23,24,25,26,29,30,31,33]. Globally, lack of awareness about early identification of hearing loss poses challenges to effective NHS implementation. For example, only 22% of parents in the United States are aware of the appropriate next steps after their child receives a hearing loss diagnosis [70]. Investigating effective methods to educate parents about the importance of early hearing screening and the potential consequences of delayed intervention is necessary. This could include developing culturally appropriate educational materials and community outreach programs. Healthcare providers’ knowledge is also limited as reported by Saudi studies [27,28,32]. For example, nurses are the primary healthcare practitioners who perform NHS [71]; however, they do not receive training on hearing loss, its prevention, its impact on speech and language development, or the importance of referring patients for diagnostic testing [72,73]. The current knowledge gaps among healthcare providers regarding NHS require developing targeted training programs to enhance their understanding and engagement in the screening process.

Both in Saudi Arabia and worldwide, ensuring that infants who fail initial screenings receive timely follow-up assessments is a significant hurdle. In Saudi Arabia, the lack of parental knowledge about follow-up recommendations and the importance of NHS is a common reason for defaults [18,25]. Similarly, global reports indicate that delays and loss to follow-up are prevalent issues, raising concerns about achieving acceptable screening coverage [34,74]. High rates of lost to follow-up significantly compromise the effectiveness of NHS programs. Conducting qualitative studies to understand the specific reasons for lost to follow-up in different populations, considering factors such as cultural beliefs, socioeconomic status, and healthcare accessibility, is important. Interventions aimed at reducing lost to follow-up, such as reminder systems, transportation assistance, and flexible scheduling of follow-up appointments, require development and evaluation.

Effective data management is crucial for monitoring the progress of infants through the NHS process. Inadequate data entry and tracking systems can lead to infants being lost to follow-up and documentation, where their screening status becomes unknown, undermining the program’s effectiveness. In Saudi Arabia, studies have shown deficiencies in data entry, management, and tracking systems which pose significant challenges to the NHS programs [18,19,23,25]. Globally, no tracking systems for infants who were either not screened or did not pass the screening and required audiological evaluation and treatment were reported [51]. The current data management systems used in NHS programs need assessments to identify weaknesses and areas for improvement and explore the development and implementation of robust, user-friendly electronic health record systems that facilitate accurate data entry and real-time tracking of patients through the screening and follow-up process.

Disparities in healthcare infrastructure can result in limited access to NHS services in certain residential areas, particularly in rural or underserved regions. The coverage rate of NHS programs in Saudi Arabia was reported between 92.6 and 96% across Saudi [19,23]. Different coverage rates were reported worldwide. For instance, half of the European countries have implemented nationwide EHDI programs, while nearly all countries in the Southeast Asia region have yet to establish NHS programs [51]. This lack of access can lead to delays in screening and intervention, adversely affecting child development. Studies to map the availability of NHS services, identify regions with limited access, and evaluate the feasibility and effectiveness of deploying mobile hearing screening units to reach underserved areas are suggested.

The quality of NHS programs heavily relies on the expertise of the personnel conducting the screenings. Insufficient training and high staff turnover can lead to inconsistencies in screening practices and affect program outcomes. The included studies recognized the prevailing lack of adequate training, especially in effective counseling for NHS personnel, along with the frequent turnover of NHS staff [18,19,33]. This is consistent with frequently lacking awareness of different screening protocols, referral procedures, hearing loss risk factors, and available resources among healthcare practitioners worldwide, which may stem from insufficient training [62,63,64,65,66]. Developing and assessing standardized training curricula for NHS personnel to ensure consistent and high-quality screenings are recommended. Furthermore, it is advised to investigate factors contributing to staff turnover in NHS programs and develop strategies to improve job satisfaction and retention.

Both in Saudi Arabia and worldwide, variations in screening protocols can lead to inconsistencies in identifying and managing hearing impairments [18,19,75]. The absence of standardized guidelines may result in disparities in care and outcomes. Collaboration with stakeholders should be carried out to develop evidence-based, standardized NHS protocols that can be adapted to different healthcare settings. The effectiveness of standardized protocols in various contexts should be studied and barriers to their adoption identified. By addressing these challenges through targeted research and interventions, NHS programs can be optimized to ensure early detection and intervention for all infants, thereby supporting their developmental potential.

### 4.3. The Main Limitations of the Included Studies

The main limitations of all included studies in this review were related to not specifically investigating the challenges of the NHS programs. Furthermore, specific limitations to each included study were identified. For example, parents were asked to provide information about their child’s hearing and neonatal hearing screening which may introduce recall bias [26]. Seven studies gathered data through self-administered questionnaires, relying primarily on the participants’ honesty and personal perceptions, which could have led to inaccurate responses [24,26,27,28,29,30,32]. The small sample size was one of the limitations of the included studies [27,28,29,32,33]. The quality of all included studies was low based on their study designs; however, the variation between studies based on study sample, sample size, location of the study sample, study purpose, and study method plays a more significant role in shaping the conclusions than the strength of evidence alone. Specifically, those studies categorized as having lower strength of evidence did not systematically introduce bias in a way that would undermine the overall findings. That said, study designs of the included studies highlight the need for more rigorous, higher-quality studies with strong methodologies and research designs to be implemented in future research. Research is also needed to further investigate the challenges that face the NHS programs in governmental and non-governmental hospitals in Saudi Arabia.

### 4.4. Limitations of the Current Review

Despite our best efforts, some study limitations were inevitable. Not all databases were accessible for searching for eligible studies. However, additional sources were checked for any research that the first search strategy might have overlooked. The inclusion criteria were open to all types of evidence, so weak study design studies were included in the review. Another drawback of the inclusion criteria was the inclusion of only studies published in and after 2016. The authors provided relevant research findings for each challenge factor; however, the study did not include an analysis of correlations between these factors.

## 5. Conclusions

The current systematic review explored the challenges of the NHS programs in Saudi Arabia. The included studies were either expert opinion, survey, or database studies. Several challenges were identified. The most common challenge was the lack of awareness and gap in knowledge among parents and healthcare professionals. National education campaigns about the NHS program and the EHDI services are critically needed. Lost to follow-up is a major challenge that requires a dedicated multidisciplinary team, a focus on public awareness, and improved documentation by using suitable database management and tracking systems. This review also identified the need for expanding the EHDI services for children in all regions of Saudi Arabia. The results obtained from the present review may assist in overcoming these challenges and improving the NHS programs in Saudi Arabia.

## Figures and Tables

**Table 1:** Number of hits for each database after duplicate removal.

Database	Total
Arab World Research Source	0
CINAHL via EBSCOhost	0
Cochrane Central	0
Embase	0
Google Scholar	14
PubMed	13
ScienceDirect	0
**Total**	27

**Table 2:** Characteristics of included studies.

Author (Year)	Study Language	Study Sample	Sample Size	Location of the Study Sample	Study Purpose	Study Method
Alanazi (2020) [18]	English	Newborns	20,171 newborns	Riyadh	Examine the referral and lost to follow-up rates of two NHS programs	Retrospective study: Retrieved NHS data from the registries in two main hospitals
Alaql (2021) [19]	English	Newborns	>1 million newborns	All regions	Review the status of the NHS national program	Expert opinion: Scientific view about the NHS national program based on a review of scientific evidence
Alothman et al. (2024) [23]	English	Newborns	199,034 newborns (147 MOH birthing hospitals)	All regions	Evaluate screening coverage rates, referral/fail rates, and follow-up procedures	Retrospective study: Retrieved NHS data from the national registry
Khurayzi et al. (2024) [24]	English	Parents	1253 parents	All regions	Examine parental knowledge with attitudes toward childhood hearing loss, and available hearing services	Prospective study: A validated questionnaire was used to achieve the aim of the study
Alothman et al. (2023) [25]	English	Newborns Parents	2312 newborns 424 parents	Riyadh	Explore the rate of follow-up default and possible reasons in a hospital-based NHS program	- Retrospective study: Retrieved NHS data from a tertiary hospital-based NHS program - Prospective study: Parents of all newborns who defaulted the follow-up on screening or diagnosis were interviewed
Almatrafi et al. (2023) [26]	English	Parents	1533 parents	All regions	Examine parents’ perceptions of NHS and identify predictors for NHS recall	Prospective study: A validated questionnaire was used to achieve the aim of the study
Malas et al. (2022) [27]	English	Pediatricians	67 pediatricians	Jeddah	Investigate knowledge and attitude of NHS and management of hearing among pediatricians in a single tertiary academic healthcare institution	Prospective study: A validated questionnaire was used to achieve the aim of study in a single tertiary academic healthcare institution
Alqudah et al. (2021) [28]	English	Family physicians	133 family physicians	All regions	Evaluate family physicians’ knowledge, attitudes, and practices related to hearing loss in children	Prospective study: A validated questionnaire was used to achieve the aim of the study
Alsudays et al. (2020) [29]	English	Parents	243 parents	Qassim	Explore parents’ knowledge and attitudes regarding childhood hearing loss and hearing services	Prospective study: A validated questionnaire was used to achieve the aim of the study in five medical centers
Al-Yahya et al. (2020) [30]	English	Mothers	384 mothers	Al-Ahsa	Examine maternal knowledge and attitudes regarding the risk factors, early detection, and early intervention of neonatal hearing loss	Prospective study: A validated questionnaire was used to achieve the aim of study in a maternity and children’s hospital
Alkahtani et al. (2019) [31]	English	Children Caregivers	1166 children 174 Caregivers	Riyadh and Dammam	Explore the average age of identification and characteristics of Saudi children with sensorineural hearing loss	- Retrospective study: Retrieved data of children aged 0–10 years old from audiology clinics in four hospitals in Riyadh and Dammam - Prospective study: Caregivers of 0–12-year-old children who visited audiology clinics in four hospitals in Riyadh were surveyed.
Almutairi et al. (2019) [32]	English	Pediatricians	216 pediatricians	All regions	Examine the knowledge, attitude, and practices of Saudi pediatricians regarding NHS	Prospective study: A validated questionnaire was used to achieve the aim of the study in 54 hospitals
Alyami et al. (2016) [33]	English	Parents	60 parents	Riyadh	Explore the status of early intervention services provided to children who aged 0–5 years and are deaf or hard of hearing and their parents	Prospective study: A semi-structured interview using a validated questionnaire was used to achieve the aim of the study in two hospitals

Note. NHS: Newborn hearing screening; MOH: Ministry of Health.

**Table 3:** Research design and strength of evidence of included studies.

Reference	Study Design	Strength of Evidence
Alanazi (2020) [18]	Cross-sectional retrospective descriptive study	Low
Alaql (2021) [19]	Expert opinion	Low
Alothman et al. (2024) [23]	Cross-sectional retrospective descriptive study	Low
Khurayzi et al. (2024) [24]	Cross-sectional prospective descriptive study	Low
Alothman et al. (2023) [25]	Study 1: Cross-sectional retrospective descriptive study Study 2: Cross-sectional prospective descriptive stud	Low
Almatrafi et al. (2023) [26]	Cross-sectional prospective descriptive study	Low
Malas et al. (2022) [27]	Cross-sectional prospective descriptive study	Low
Alqudah et al. (2021) [28]	Cross-sectional prospective descriptive study	Low
Alsudays et al. (2020) [29]	Cross-sectional prospective descriptive study	Low
Al-Yahya et al. (2020) [30]	Cross-sectional prospective descriptive study	Low
Alkahtani et al. (2019) [31]	Study 1: Cross-sectional retrospective descriptive study Study 2: Cross-sectional prospective descriptive stud	Low
Almutairi et al. (2019) [32]	Cross-sectional prospective descriptive study	Low
Alyami et al. (2016) [33]	Cross-sectional prospective descriptive study	Low

**Table 4:** Challenges of the NHS programs in Saudi Arabia.

Reference	Challenges of the NHS Programs
Alanazi (2020) [18]	High lost to system rate (lost to follow-up and lost to documentation rate) Lack of parental awareness of the importance of NHS Non-standardized NHS protocols The absence of a unified data management and tracking system among all governmental and private hospitals Lack of automated transfer of data from the screeners to the database without being manually inputted Insufficient training for NHS personnel specifically training on effective counseling
Alaql (2021) [19]	Frequent changes in the NHS team, especially trained nurses due to a high turnover rate Lack of automated data entry which may lead to human errors Limited attention to monitoring refers and lost to follow-up cases Delay of access to appropriate support for diagnosis and intervention Need for more educational programs for both the parents and healthcare professionals
Alothman et al. (2024) [23]	Lack of documentation of lost to follow-up newborns The diagnostic stage is not included in the NHS national registry NHS data of non-MOH and private hospitals is not included in the NHS registry Limited public awareness of the NHS
Khurayzi et al. (2024) [24]	Low level of parental knowledge about the NHS program and hearing loss identification sooner after birth
Alothman et al. (2023) [25]	High lost to follow-up rate due to several reasons: Lack of awareness regarding the recommended follow-up screening Parental and health issues (e.g., failure to remember, parental health conditions, Coronavirus disease, etc.) Logistical issues (e.g., no transportation and work commitments) Need for implementing a tracking system
Almatrafi et al. (2023) [26]	Inadequate awareness of NHS among parents
Malas et al. (2022) [27]	Gaps in knowledge and attitude about NHS and management of hearing loss among pediatricians
Alqudah et al. (2021) [28]	Unsatisfactory knowledge of hearing loss, including assessments and the presence of the national early hearing detection and intervention program
Alsudays et al. (2020) [29]	Poor parental knowledge about childhood hearing loss including identification and intervention
Al-Yahya et al. (2020) [30]	Low maternal awareness of the early identification and management of hearing loss
Alkahtani et al. (2019) [31]	No nationwide covering of NHS program Lack of parental knowledge about sensorineural hearing loss even if they may be suspicious about their child’s hearing
Almutairi et al. (2019) [32]	Gaps in knowledge and attitude about NHS
Alyami et al. (2016) [33]	Information gaps about early intervention Poor communication among service providers Lack of services in some residential areas Need for financial support to cover costs (e.g., travel costs) and social support

## Data Availability

Data are contained within the article.

## Appendix A

**Table A1:** Search terms, databases, and search strings.

Search Terms	Database	Search Strings
Population: newborns, neonates, infants Type: hearing screening in Saudi Arabia Outcomes: challenges, difficulties, threats	PubMed and CINAHL^®^ database searches consisted of MeSH term/CINAHL	Heading newborns (OR) neonates (OR) infants is a major topic combined (AND) with the terms hearing screening (AND) Saudi Arabia. The citations retrieved from this search were further narrowed using the terms (challenges (OR) difficulties (OR) threats).

Note. CINAHL = Cumulative Index to Nursing and Allied Health Literature; MeSH = Medical Subject Heading.
